# Flexible and Transparent Circularly Polarized Patch Antenna for Reliable Unobtrusive Wearable Wireless Communications

**DOI:** 10.3390/s22031276

**Published:** 2022-02-08

**Authors:** Abu Sadat Md. Sayem, Roy B. V. B. Simorangkir, Karu P. Esselle, Ali Lalbakhsh, Dinesh R. Gawade, Brendan O’Flynn, John L. Buckley

**Affiliations:** 1 School of Electrical & Data Engineering, University of Technology Sydney, Sydney, NSW 2007, Australia; karu.esselle@uts.edu.au; 2 School of Engineering, Macquarie University, Ryde, NSW 2109, Australia; ali.lalbakhsh@mq.edu.au; 3 Tyndall National Institute, T12 R5CP Cork, Ireland; roy.simorangkir@ieee.org (R.B.V.B.S.); dinesh.gawade@tyndall.ie (D.R.G.); brendan.oflynn@tyndall.ie (B.O.); john.buckley@tyndall.ie (J.L.B.)

**Keywords:** circular polarization, flexible, polymer, transparent, wearable

## Abstract

This paper presents a circularly polarized flexible and transparent circular patch antenna suitable for body-worn wireless-communications. Circular polarization is highly beneficial in wearable wireless communications, where antennas, as a key component of the RF front-end, operate in dynamic environments, such as the human body. The demonstrated antenna is realized with highly flexible, robust and transparent conductive-fabric-polymer composite. The performance of the explored flexible-transparent antenna is also compared with its non-transparent counterpart manufactured with non-transparent conductive fabric. This comparison further demonstrates the suitability of the proposed materials for the target unobtrusive wearable applications. Detailed numerical and experimental investigations are explored in this paper to verify the proposed design. Moreover, the compatibility of the antenna in wearable applications is evaluated by testing the performance on a forearm phantom and calculating the specific absorption rate (SAR).

## 1. Introduction

With the rapid advancement of flexible materials and manufacturing technologies, new opportunities for flexible electronics are emerging. The flexible electronics industry is worth a billion-dollar market value worldwide. A flexible antenna is considered as the most prominent invention in the flexible electronics industry. The applications of flexible antennas have spanned beyond the realm of traditional wireless communications. Nowadays, antennas have become an indispensable component in body centric wireless communications, remote sensing technology, national surveillance and security services, battle fields, firefighting and rescue missions, sports and fitness and vehicular communications. These applications have greatly benefited from flexible, robust, low-profile and small antennas owing to their flexible deployment on the system platforms, regardless of the curvature of the surface, thus maximizing the use of existing infrastructure, as well as their capability to withstand physical deformation and rigorous environmental circumstances. Apart from flexibility and robustness, unobtrusiveness is also a demanding characteristic of antennas in many applications not only for enhancing aesthetics but also for ensuring the reliability of operations. One example is wireless wearable technology [1,2], which is used in the healthcare and well-being sector, including remote sensing, monitoring and detection of patients suffering from chronic and fatal diseases, isolated communities, mental health patients, aged people and even contagious diseases like COVID-19. To ensure efficient sensing and monitoring performance of a wearable wireless healthcare system, antennas, as one of the enabling technologies, need to be comfortable, easy to wear, and visually imperceptible so that they impose minimum interference with daily activities.

Visual imperceptibility can be achieved in a number of different methods. For instance, the embroidery technique can be employed to realize an antenna embedded onto a patients’ outfit, resulting in very little visual appearance. The major drawback is that, after repeated washing, the antenna performance is significantly degraded [3,4], thus affecting its long-term usage. Another approach is by hiding the antenna inside the wearer’s outfit. This could possibly be the handiest way to make an antenna’s appearance entirely invisible. This approach, however, implies that the antenna performance is significantly impacted by the dielectric properties of the outfit materials [5,6]. Such effect is severe, particularly upon the outfit’s exposure to water (e.g., from the wearers’ sweat or rain). As an alternative to these approaches, antennas’ appearance can be made imperceptible by making the antennas optically transparent. In a wearable environment, using a robust, flexible and optically transparent antenna is the most effective solution for setting up an imperceptible communication network [7]. In addition to body centric communications, flexible-transparent antennas have a number of other potential applications, such as vehicular communications [8], concealed network terminals, smart cities [9] and solar cells, where antennas that are optically transparent and can be mounted on curved surfaces are desirable. However, the development of flexible and transparent antennas entirely depends on unconventional materials [10]. The unavailability of competent materials has obstructed the progress of flexible-transparent antennas’ development.

To the best of our knowledge, there have been quite a few efforts that have succeeded in demonstrating antennas that are simultaneously flexible and transparent. The unavailability of perfect transparent conductors is one of the possible reasons of this scarcity. Transparent conductors used in antenna fabrication are broadly divided into two categories, i.e., thin films and meshed conductors; both groups are associated with some limitations. The group of thin film conductors includes indium-tin-oxide (ITO) [11], multi-layer ITO (IZTO/Ag/IZTO) film [12], fluorine-doped tin oxide (FTO) [13], gallium-doped zinc oxide (GZO) [14] and silver-coated polyester (AgHT-8) film [15]. In general, these conductors exhibit poor conductivity. Moreover, the common thin films, such as ITO [11], FTO [13] and GZO [14], are fragile, hence not compatible for conformal applications. Some approaches, however, have been reported to make them flexible. For example, in [12], Zn was mixed with an ITO film to increase its flexibility. Furthermore, a stacked topology of IZTO films comprising two layers of IZTO with Ag in between was introduced to increase the conductivity. Despite the flexibility of IZTO/Ag/IZTO film, it has 4.99 Ω/sq sheet resistance, which is quite high for an efficient antenna operation. The second group, meshed conductors, on the other hand, achieves transparency by incorporating perforations throughout the surface of traditional conductive sheets [16,17]. The transparency of meshed conductors depends on the ratio of opening to the total surface area. The larger the opening on the conductor surface, the higher the transparency that can be achieved. This unfortunately results in an increase of sheet resistance subsequently. On top of that, special treatment is required to integrate meshed conductors to flexible substrates to realize flexible transparent antennas [18]. It should also be noted that both groups of transparent conductors require strictly optimized fabrication processes to achieve the right density of the conductive structure for a balance between the conductivity performance and transparency [9], which are generally complex, cumbersome, and costly [19]. This might also result in reproducibility issues of an antenna with a desired performance. Using polymer and additive manufacturing, flexible, transparent and stretchable structures can be achieved, which is successfully demonstrated by [20,21,22]. Recently, a new technology has been reported that has combined commercially available transparent conductive fabric with solution-processable polydimethylsiloxane (PDMS) [7,23,24]. This technology is relatively simple, effective, cost-efficient and reproducible for realizing robust-flexible-transparent antennas. This technology is utilized in this work to fabricate the proposed circularly polarized (CP) antenna.

Circular polarization is an important property of antennas in many applications, especially when they are mounted on moving objects, such as in the case of wearable applications. CP antennas are less impacted by multi-path effects and polarization mismatch losses than linearly polarized (LP) antennas [8,25]; thus, CP antennas can enhance the reliability of wireless communication links. By avoiding the power loss due to polarization mismatch, CP antennas also play an essential role in extending the battery life, which is often a concern and limiting factor in low-power wireless communication systems [26]. In this paper, a flexible and transparent single-feed CP microstrip patch antenna is designed. The explored design incorporates a rectangular slot at the middle of the patch and a chamfer at the patch periphery to achieve CP radiation. While significant efforts in developing CP wearable antennas have been reported in recent years [27,28,29,30,31,32,33,34,35,36,37,38], to our knowledge, this is the first CP wearable antenna ever reported that is concurrently flexible and transparent.

The design process, fabrication methods and numerical and experimental investigations of the proposed flexible and transparent CP antenna are demonstrated in this paper. The proposed antenna operates at 2.4 GHz Industrial, Scientific and Medical (ISM) band that has a number of applications including wireless body area network (WBAN). The target application of the antenna is wearable technology. To evaluate its suitability in wearable applications, specific absorption rate (SAR) is calculated in a forearm phantom. Two antenna prototypes are fabricated, one with transparent fabric and another one with non-transparent fabric, as a reference. It is worth mentioning that a comparative study between flexible antennas based on transparent and non-transparent conductive fabric has never been reported before. This investigation will be an important source of information to understand the potential of the proposed materials for the development of robust, flexible, and unobtrusive wearable antennas.

## 2. Materials

The most crucial step in the development of flexible-transparent antennas is the selection of appropriate materials. The development of flexible antennas’ entirety depends on unconventional materials where the selected conductive and dielectric materials should have high durability against physical deformations, endurance to environmental impacts, easy availability, low-cost, simple fabrication process, high optical transparency and low-loss. These properties are offered by the transparent conductive fabric-PDMS composite, for which reason it is used to fabricate the CP antenna proposed in this work.

The conductive parts of the demonstrated antenna (i.e., the patch, feed-line, and ground plane) are fabricated with transparent conductive fabric, VeilShield, developed by Less EMF Inc., Latham, NY, USA. VeilShield is an ultra lightweight, thin (57 μm), flexible and corrosion resistant mesh style conductive fabric. The mesh is formed by intertwining monofilament polyester threads, with 132/inch mesh accomplishing nearly 72% optical transparency. The polyester threads are coated with Nickel/Zinc blackened Copper, which makes the fabric conductive with 0.1 Ω/sq sheet resistance. This transparent conductive material benefits from its availability in the form of a fabric sheet with structural regularity. As a result, the electrical performance and optical transparency are more predictable and stable, which helps in solving the reproducibility issue encountered with other types of transparent conductive materials.

As the substrate and encapsulation of the antenna, PDMS, which is a mineral-organic elastomeric polymer constituting carbon and silicon, is used. PDMS exhibits some unique properties that make it emerging for flexible electronics fabrication. This includes high transparency (>94% [39]), high flexibility, waterproof, and biocompatibility. Another notable advantage of PDMS is its simple processing. The PDMS solution, a mixture between the base and cross-linking agent (curing agent), can be cured even in room temperature and does not necessitate clean room-based treatment. Due to its initial liquid form, once poured into customized mold and cured, nearly any shape and thickness of the flexible substrate can be achieved. Based on the measurement conducted with an Agilent 85070E Dielectric Measurement Kit, PDMS exhibits a relatively constant permittivity of 2.75 from 0.5–10.6 GHz with a frequency dependent loss tangent ranging from 0.008 to 0.07. In this work, the PDMS solution is made with Dow Corning Sylgard 184 silicone elastomer kit with base to cross-linking agent mixing ratio of 10:1. PDMS makes extremely strong integration with conductive fabric, thanks to the percolation of PDMS solution into the mesh, which leads to strong bonding and seals the fabric firmly upon curing [7,40]. To take into account the portion of PDMS penetration into the mesh, an effective sheet resistance of 0.7 Ω/sq, obtained through intensive investigations incorporating transmission lines and T-resonator samples [7], was used to model the conductive layer in the antenna simulation process.

A non-transparent version of the proposed CP antenna design was also fabricated as a reference. The utilized non-transparent conductive fabric is nickel–copper coated ripstop which has a thickness of 0.08 mm and sheet resistance of 0.03 Ω/sq. Similar to the transparent conductive fabric, the PDMS percolation into this fabric was taken into account during the antenna design process by using an adjusted sheet resistance of 0.23 Ω/sq [40] to model the antenna conductive parts.

## 3. Antenna Topology and Design

### 3.1. Antenna Configuration

**Figure 1:**
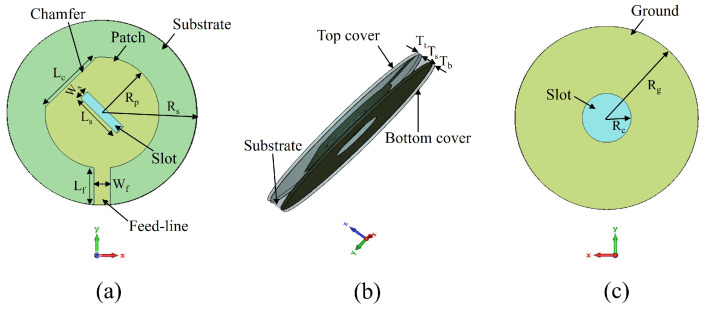
Geometry of the proposed flexible and transparent CP antenna: (**a**) top view, (**b**) side view, (**c**) bottom view.

The topology of the proposed flexible and transparent circularly-polarized antenna is shown in Figure 1. The demonstrated antenna is a microstrip circular patch antenna with a rectangular central slot and a chamfer at the edge. On the opposite side of the patch, there is a circular ground plane with a circular slot etched at the center. To ensure protection against humidity, dust, chemical and harsh bending scenarios, the antenna is encapsulated by PDMS. Very thin encapsulation is used to avoid RF performance degradation.

The numerical modelling and optimization of the proposed antenna were conducted in CST Microwave Studio 2020 that uses Finite Integration Technique (FIT) for solving Maxwell’s electromagnetic equations. The optimized dimensions of the antenna are shown in Table 1.

### 3.2. Design Methodology

**Figure 2:**
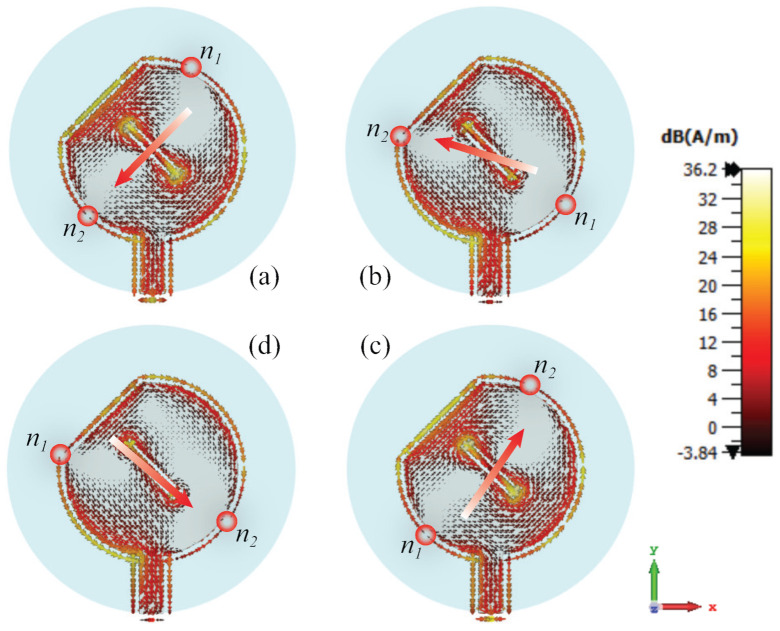
Surface current distribution of the antenna at 2.4 GHz at the time phases (**a**) 0°, (**b**) 90°, (**c**) 180° and (**d**) 270°.

The CP characteristic of the antenna is achieved by exciting two orthogonal modes having the same magnitudes but with 90° phase difference [36]. The proposed circular-shaped antenna operates at its fundamental TM_11_ mode. The rectangular slot at the center of the patch and the chamfer at patch periphery concurrently degenerate the fundamental TM_11_ mode into two orthogonal modes that produce CP [36]. The dimensions of the slot and chamfer are optimized to maintain the same magnitudes and phase quadrature of the degenerated orthogonal modes (E_*x*_ and E_*y*_). The slot and chamfer drive the surface current to rotate 90° at each time phase quadrature, which is the ideal characteristic of the CP wave. To observe the CP characteristic of the radiated wave of the antenna, its surface current distribution is analysed. Figure 2 illustrates the surface current distribution of the antenna at 2.4 GHz as the feeding phase progresses from 0° to 270° by 90° interval. For ease of observation, we added labels n_1_ and n_2_, each of which denotes the null location of the current distribution and marked accordingly the tendency of the current vector direction with an arrow. It can be seen from Figure 2 that the current vector of the antenna rotates clockwise as the phase progressing, which indicates a left-handed circular polarization in the direction of the +*z*-axis.

Due to the loss associated with the materials used, the antenna suffers from low efficiency and gain. A defected ground plane approach was therefore implemented by cutting a circular slot at the center of the ground to improve the gain and efficiency of the antenna. By cutting the slot on the ground, the overall transparency of the antenna also increases as the area where two layers of fabric overlay is reduced. However, the presence of slot in the ground plane increases the back radiation, which in turn decreases the front-to-back ratio of the radiation pattern. Further optimization of the size of the slot is, therefore, required.

**Figure 3:**
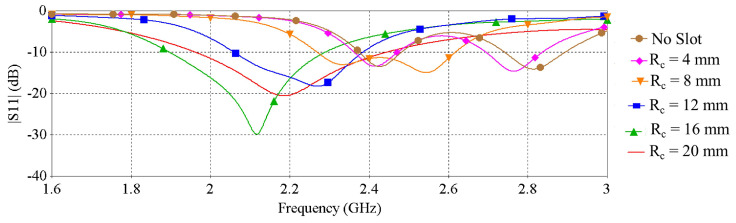
$|S_{11}|$ vs. frequency when varying the radius of the slot of the ground plane.

The effect of the radius of the slot of the ground plane on the resonance characteristics of the antenna is illustrated in Figure 3, which shows that resonance frequency shifts with the change of the slot radius.

**Figure 4:**
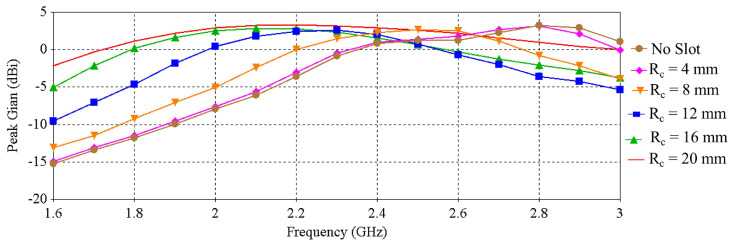
Peak gain vs. frequency when varying the radius of the slot of the ground plane.

**Figure 5:**
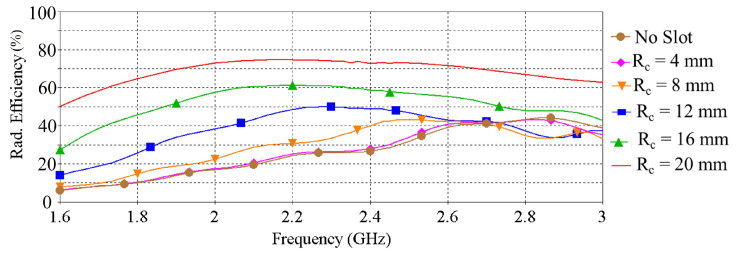
Radiation efficiency vs. frequency when varying the radius of the slot of the ground plane.

**Figure 6:**
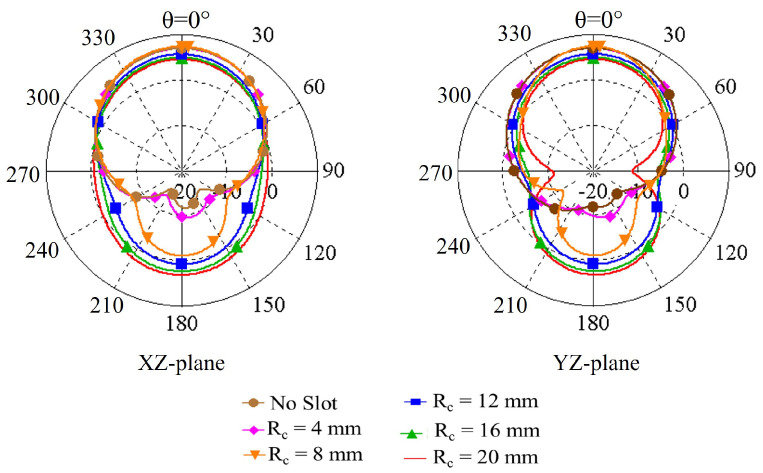
Radiation patterns (in dB) at the corresponding resonance frequencies when varying the radius of the slot of the ground plane.

The influence of the slot radius on the peak gain, radiation efficiency and radiation patterns are illustrated in Figure 4, Figure 5 and Figure 6, respectively. Figure 4 shows that the peak gain of the antenna improves significantly as the slot radius (R_*c*_) extends up to 12 mm. Beyond that point, expanding the slot radius further does not substantially enhance the gain. It can also be noticed from Figure 5 and Figure 6 that, with the increase of the slot radius, the radiation efficiency of the antenna increases as well as the back radiation. Considering the target wearable applications, the latter is not preferred as it means more radiation is directed towards the lossy human body tissue. Giving consideration to the antenna peak gain, efficiency and back radiation, the slot radius of 8 mm was selected in this design, which provides a maximum peak gain of 2.67 dBi, maximum radiation efficiency of nearly 43.2% and front-to-back ratio of 8.6 dB. The findings of this investigation are summarized in Table 2.

**Figure 7:**
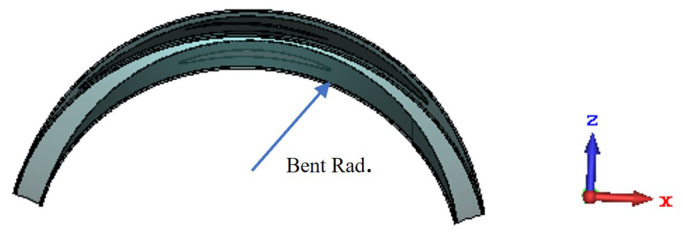
The antenna topology under bending.

**Figure 8:**
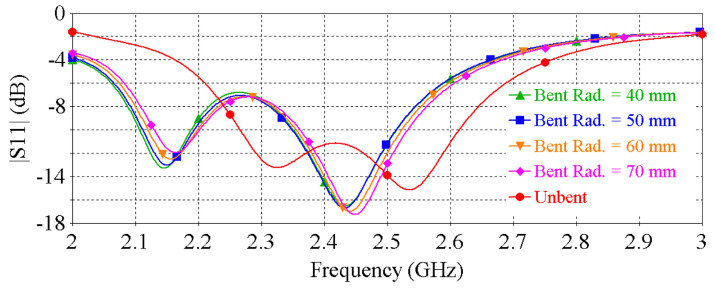
$|S_{11}|$ vs. frequency of the antenna for different bending radius.

**Figure 9:**
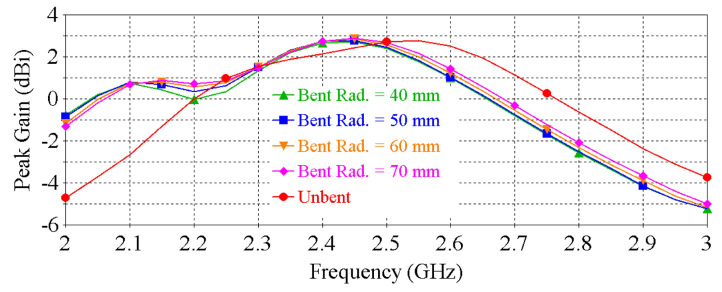
Peak gain vs. frequency of the antenna for different bending radius.

The demonstrated antenna is conformal, which means that it can undergo bending that is crucial for wearable applications. To assess the RF performance of the antenna under bending, the antenna was simulated when bent as shown in Figure 7. “Bent Rad.” denotes the bending radius of the antenna. Figure 8 and Figure 9 show the $|S_{11}|$ and peak gain of the antenna for different bending radii ranging from 40 to 70 mm. It can be noted that the resonance frequency of the antenna is shifted slightly towards lower frequency as the bending radius decreases. Nevertheless, the target frequency of 2.4 GHz is still covered with a good matching and satisfactory gain. These numerical investigations show that the antenna is resilient against physical deformation, validating the suitability of the antenna for wearable applications.

**Figure 10:**
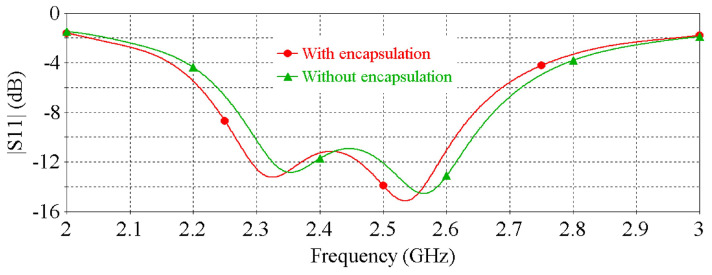
The effect of the PDMS encapsulation on the $|S_{11}|$ of the antenna.

**Figure 11:**
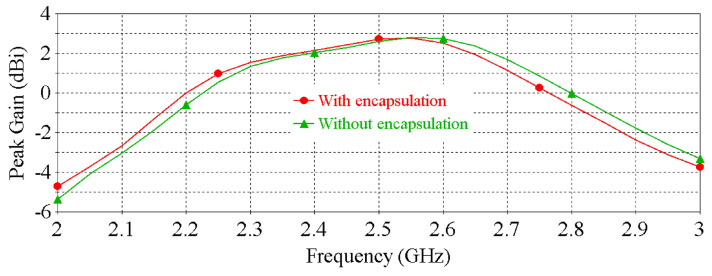
The effect of the PDMS encapsulation on the peak gain of the antenna.

Lastly, Figure 10 and Figure 11 show the impacts of PDMS encapsulation on the resonance frequency and peak gain of the antenna, respectively, demonstrating that the thin (0.2 mm) PDMS encapsulation has an insignificant effect on the antenna performance.

## 4. Prototype Fabrication

**Figure 12:**
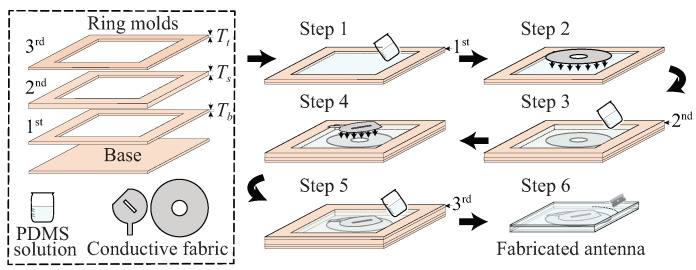
Schematic representation of the antenna fabrication process.

The demonstrated transparent and non-transparent flexible antennas are fabricated by utilizing a cost-effective, straightforward and environmental-friendly method. It can be noted that the traditional physical vapor deposition [12], photo-lithography [22,41], RF sputtering [11], spray pyrolysis [13] and inkjet printing [42] methods of antenna manufacturing are often associated with fabrication complexity, high cost and involvement of hazardous chemicals and toxic by-products. In contrast to these methods, our demonstrated method is significantly cost-effective, simple and free from producing toxic chemicals. In the explored method, conductive fabric is used as the conductive parts of the antenna. Conductive fabric is certainly easy to handle, and it is easy to maintain structural regularity that is often challenging in metal deposition techniques. Moreover, the substrate is made with elastomer polymer, PDMS, which can be shaped to any pre-defined thickness by customized molds. This feature exhibits manufacturing flexibility and a simple method of maintaining accuracy. In principle, the antenna fabrication procedure is similar to our previously demonstrated works in [7,40,43]. The major difference is that, in this work, the conductive fabrics were patterned using a cutting machine, rather than through a manual cutting process with a razor blade. We found this crucial as the CP characteristic is typically very sensitive to the variation of the patch dimension. The schematic representation of the antenna fabrication process is shown in Figure 12.

Below, the details of the manufacturing process are provided.

**Detailed process:** The antennas were fabricated by adopting a systemic procedure as described below:

**Step-1:** The first mold having the thickness 0.2 mm was attached to a base PCB board using sealant. Then, the PDMS solution was poured into the mold to create the bottom encapsulation layer. When required (e.g., if there are a lot of bubbles trapped in the PDMS solution), a vacuum desiccator can be used with approximately −80 kPa pressure to suck the air bubbles out. The mold with PDMS solution was kept in an oven at 80 °C for 30 min for curing.

**Step-2:** The ground plane was attached on top of the cured bottom PDMS cover, built in step 1, with small amount of uncured PDMS. Then, the molds were kept in an oven at 80 °C for 30 min for curing the attachment.

**Step-3:** The second mold of 3 mm thickness was attached on top of the first mold with sealant. The PDMS solution was poured into the second mold, filling up to the thickness of the mold. Then, the desiccation and curing procedures applied in step 1 were repeated to build the substrate layer of the antenna.

**Step-4:** The patch was attached on top of the cured substrate PDMS layer, built from step 3, with a small amount of uncured PDMS. Then, step 2 was followed to properly attach the patch on top of the substrate.

**Step-5:** The third mold of 0.2 mm thickness was attached on top of the second mold with sealant. The PDMS solution was poured into the third mold up to the thickness of the mold, and the desiccation and curing procedures applied in step 1 were repeated to build the top encapsulation layer.

**Step-6:** The prototype was carefully peeled-off from the molds and the extra PDMS were trimmed to give the exact dimensions of the antenna.

**Step-7:** Small section of PDMS from the top and bottom covers were etched out to connect the SMA connector to the antenna. The pin and ground of the connector were connected to the patch and ground plane of the antenna by using a CircuitWorks conductive epoxy which was cured by applying 80 °C temperature for 10 minutes in the oven. It should be noted that conductive epoxy is the most appropriate option for connecting the SMA connector to the conductive fabric because soldering has a risk of burning the fabric, and thus is not compatible. In actual wearable implementation, more compatible connection strategies can be used, such as butterfly clasps [44], contact springs [45], snap-on button [46] or hook and loop connectors [47].

**Figure 13:**
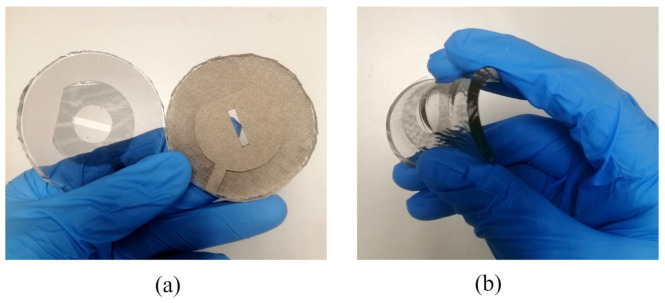
Fabricated prototypes: (**a**) transparent and non-transparent antenna prototypes, (**b**) transparent antenna in bent state.

In Figure 13, the photo of the fabricated transparent prototype is compared with its non-transparent counterpart fabricated through the similar process. As shown, the fabricated antenna exhibits high flexibility and transparency.

Three ring-shaped rectangular molds and a rectangular-shaped base were used for fabricating the antennas. Due to the availability, in this work, the molds were made from PCB boards which have the thicknesses of the target PDMS layers, cut with a slightly bigger opening than the antenna substrate aperture. The electroplated copper on the PCB was kept as it allows for an easy demolding of the built antenna at the end of the process.

PDMS solution was made with Dow Corning Sylgard 184 silicone elastomer kit. The kit comes with base silicone and curing agent. The PDMS solution was prepared by mixing the base and curing agent at the ratio of 10:1. The percentage of the curing agent in the mixture controls the flexibility level of the cured PDMS; higher percentage of the agent reduces the flexibility of the PDMS. The mixing process was undertaken in a plastic container, and the mixture was thoroughly stirred to ensure uniformity throughout the solution. The uncured PDMS solution is a clear sticky liquid, which is cured by applying heat. The curing time depends on the applied temperature.

The patch and ground plane were prepared by accurately cutting the conductive fabrics following the optimized antenna design pattern. In this work, the fabric was cut by using a cutting machine, Cricut Maker. To do this, 2D images of the patch and ground plane were exported from CST and imported into Cricut Design Space software.

## 5. Performance Investigation

**Figure 14:**
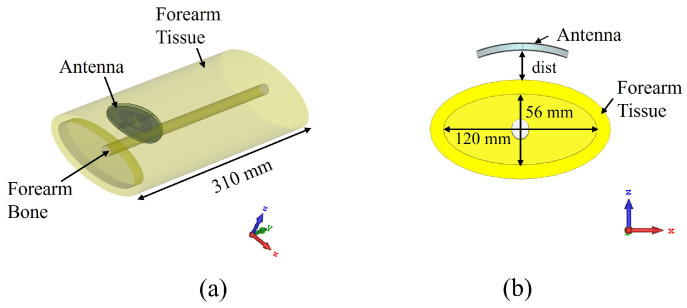
Antenna on top of the forearm phantom: (**a**) perspective view, (**b**) cross-sectional view.

The demonstrated transparent and non-transparent CP patch antennas were numerically investigated, and the prototypes were experimentally tested to explore the performance in different operating environments. The performance of the antennas was investigated both in free space and on-phantom. The latter is important to validate the antennas’ compatibility in wearable applications. In the first scenario, the antennas were investigated in the flat condition, whereas, in the second scenario, the antennas were wrapped over the phantom. On-phantom testing was conducted with SHOGFPC-V1 forearm phantom from SPEAG [45,48]. On-phantom numerical investigation was conducted by placing the antenna on top of a double layer cylinder (Figure 14) mimicking the structure of the SPEAG phantom. The forearm bone was modelled with $\epsilon_{r}$ = 30 and σ=2.5 S/m, whereas the forearm tissue was modelled with $\epsilon_{r}$ = 27 and σ=1.1 S/m, following the averaged electrical properties of the forearm at 2.4 GHz according to the SPEAG phantom data-sheet [48]. While a more complex multilayer phantom would offer a more complete understanding on the antenna SAR performance, homogeneous phantoms or phantoms with averaged properties have also been widely employed in literature [45,49,50].

**Figure 15:**
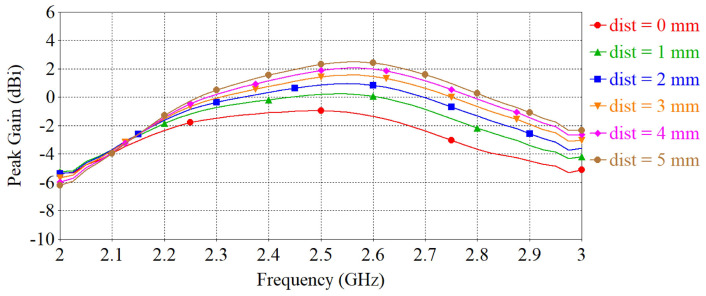
Peak gain vs. frequency when varying the separation between the antenna and phantom.

**Figure 16:**
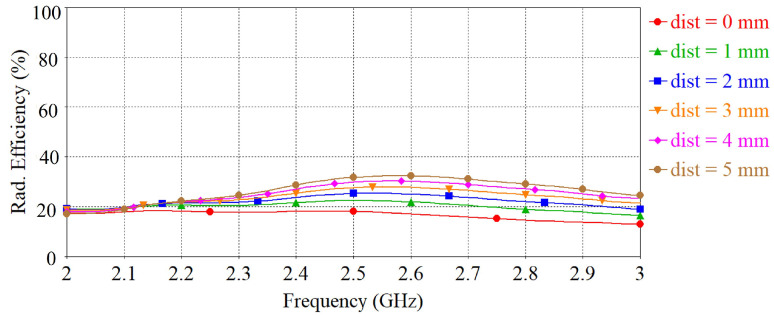
Radiation efficiency vs. frequency when varying the separation between the antenna and phantom.

**Figure 17:**
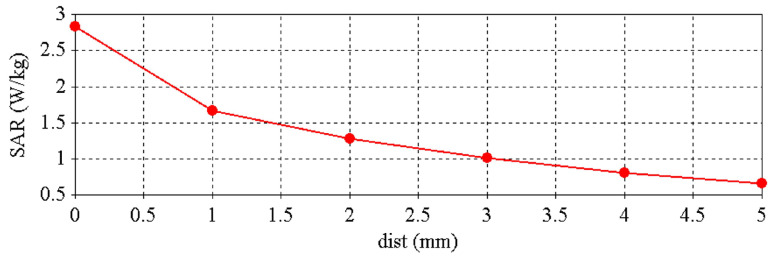
SAR at 2.4 GHz when varying the separation between the antenna and phantom.

**Figure 18:**
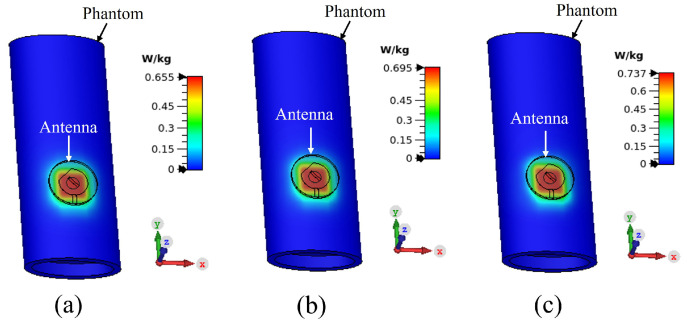
SAR distributions in the forearm phantom at: (**a**) 2.4 GHz, (**b**) 2.45 GHz and (**c**) 2.6 GHz.

**Figure 19:**
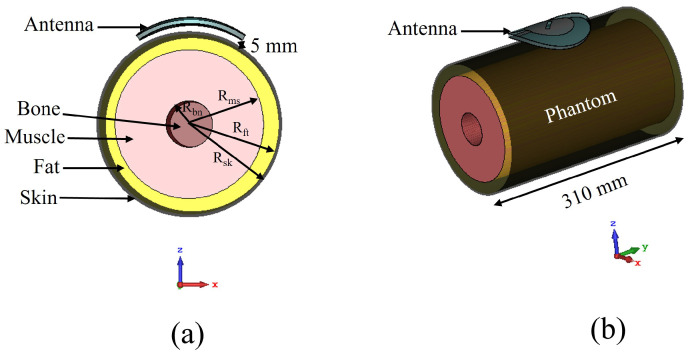
Antenna on top of the multilayer phantom: (**a**) cross-sectional view, (**b**) perspective view. (R_bn_ = 12.5 mm, R_ms_ = 40 mm, R_ft_ = 48.5 mm, R_sk_ = 50 mm).

**Figure 20:**
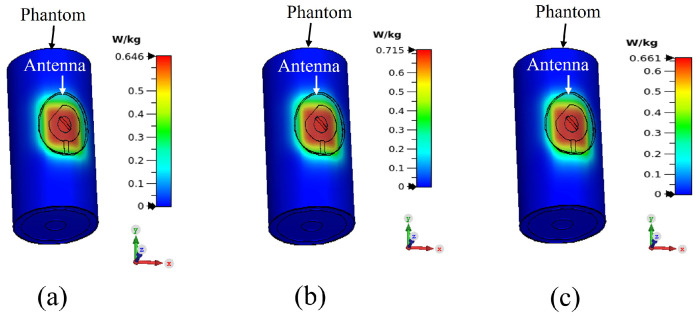
SAR distributions in the multilayer phantom at: (**a**) 2.4 GHz, (**b**) 2.45 GHz and (**c**) 2.6 GHz.

The separation between the antenna and phantom (dist) was varied and peak gain, radiation efficiency and the specific absorption rate (SAR) were calculated in simulation. The SAR was calculated by applying 0.1 W input power and averaged over 10 g of tissue. Figure 15, Figure 16 and Figure 17 depict the antenna peak gain, radiation efficiency and SAR (at 2.4 GHz), respectively, for different separation between the antenna and phantom. From these investigations, it is revealed that, when the antenna is directly mounted on phantom (dist = 0 mm), peak gain and radiation efficiency are very low but yet the SAR values are still less than the maximum permissible SAR limit for body extremities (i.e., 4 W/kg) as specified in IEEE C95.1-2019 standard [51], demonstrating that the proposed antenna can be used safely on the human body at the target 2.4 GHz ISM band. As the separation between the antenna and phantom increases, SAR decreases further and gain and radiation efficiency also improve. It should be noted that, in a real-life scenario, there will always be a gap of approximately 2 mm to 5 mm between the antenna and body by the fabrics of the wearers’ outfit and, thus, the antenna will operate well. Figure 18 shows the calculated SAR distribution in the phantom for 5 mm separation at 2.4 GHz, 2.45 GHz and 2.6 GHz. The calculated maximum 10 g-averaged SAR are 0.655 W/kg, 0.695 W/kg and 0.737 W/kg at 2.4 GHz, 2.45 GHz and 2.6 GHz, respectively. For validation, the SAR metric was further analysed by using a multilayer cylindrical phantom consisting of bone, muscle, fat and skin layers, as done in [52,53]. The configuration of the multilayer cylindrical phantom is shown in Figure 19 and the dielectric properties of each tissue layer are given in Table 3 [53]. Figure 20 illustrates the calculated SAR distribution in the multilayer cylindrical phantom for 5 mm separation at 2.4 GHz, 2.45 GHz and 2.6 GHz. The calculated maximum 10 g-averaged SAR are 0.646 W/kg, 0.715 W/kg and 0.661 W/kg at 2.4 GHz, 2.45 GHz and 2.6 GHz, respectively, which are close to the SAR values obtained with the homogeneous forearm phantom. From these investigations, it is exhibited that the ground plane provides sufficient shielding against the antenna’s back radiation, demonstrating the antenna’s suitability in wearable applications.

The performance of the antenna was experimentally tested in the worst scenario, i.e., when the antenna was directly bent around the wrist of the SPEAG forearm phantom having a circumference of approximately 15 cm. The prototype was bent in two principal planes, i.e., *x*-axis bent and *y*-axis bent. These tests were conducted for both the transparent and non-transparent antennas, and the performances were compared.

**Figure 21:**
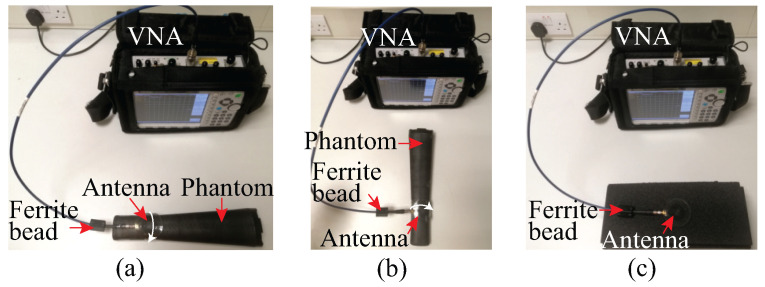
Antenna $|S_{11}|$ measurement set-up: (**a**) antenna bending towards the *x*-axis direction, (**b**) antenna bending towards the *y*-axis direction, and (**c**) flat antenna.

**Figure 22:**
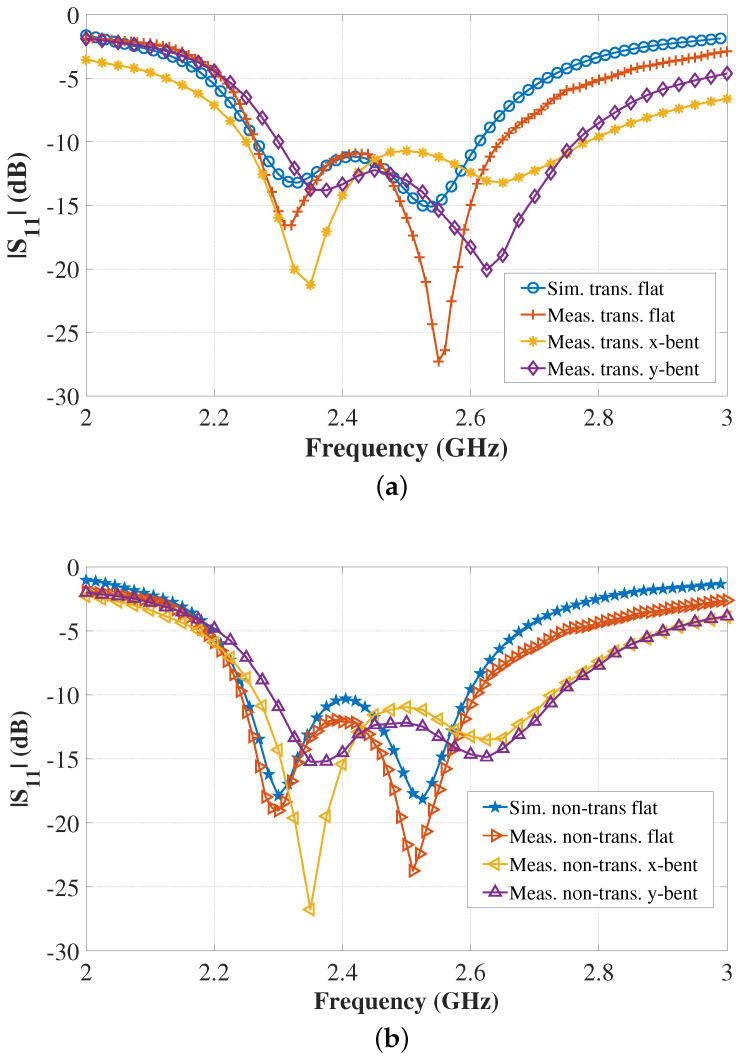
Simulated and measured $|S_{11}|$ vs. frequency: (**a**) transparent antenna, (**b**) non-transparent antenna.

The return loss ($|S_{11}|$) measurements were accomplished by using an MS2038C Vector Network Analyzer (VNA) from Anritsu. The photos of the $|S_{11}|$ measurement set-ups of the antenna are shown in Figure 21. The antenna was fed with a 50-Ω coaxial line through a 50-Ω SMA connector. A ferrite bead was incorporated into the measurement set-up to minimize the ground current flow to the feed cable, which may affect the antenna’s measured impedance and radiation characteristics. The measured and simulated $|S_{11}|$ results are displayed in Figure 22. From Figure 22, it can be seen that the measured results of the free-space case agree well with the simulated results, demonstrating a satisfactory matching ($|S_{11}|$ < 10 dB) for both transparent and non-transparent antennas at the target frequency 2.4 GHz. In simulations, as the result of the differences in the characteristics of the conductive materials, it was observed that both antennas in flat free-space case exhibit slightly different input impedance, i.e., 81.6-j17.7 Ω for the transparent antenna and 84.3-j23.5 Ω for the non-transparent counterpart. The discrepancy between the simulated and measured results are most likely due to the fabrication tolerances, such as during patch and ground plane layering over PDMS layers and the antenna-SMA interconnection using conductive epoxy. The 10-dB return-loss bandwidth is around 346 MHz for both the transparent and non-transparent antenna. It can also be observed from the on-phantom $|S_{11}|$ results in Figure 22 that the resonance frequency and impedance matching of the antennas are affected by the phantom proximity and bending. Nevertheless, the antenna still operates within the desired band with satisfactory impedance matching, which demonstrate the antenna’s robustness against human body loading and physical deformation.

**Figure 23:**
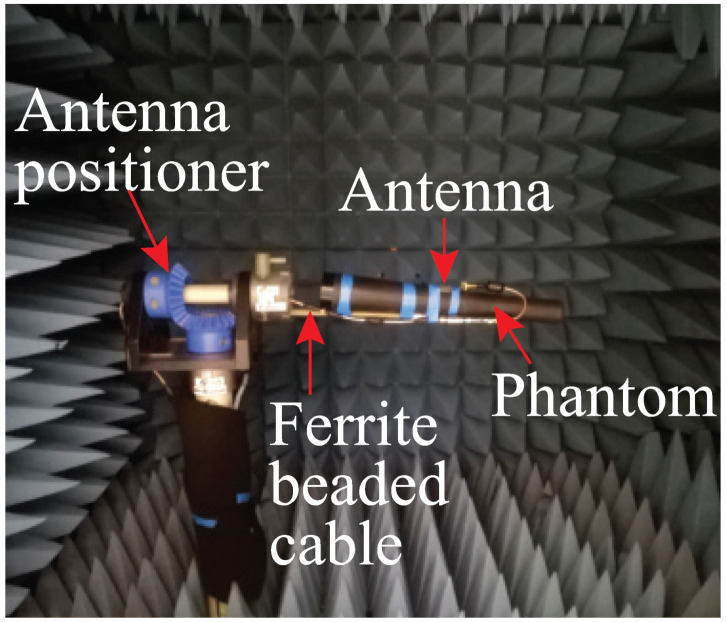
Antenna measurement set-up inside the AMS-8050 Antenna Measurement System.

**Figure 24:**
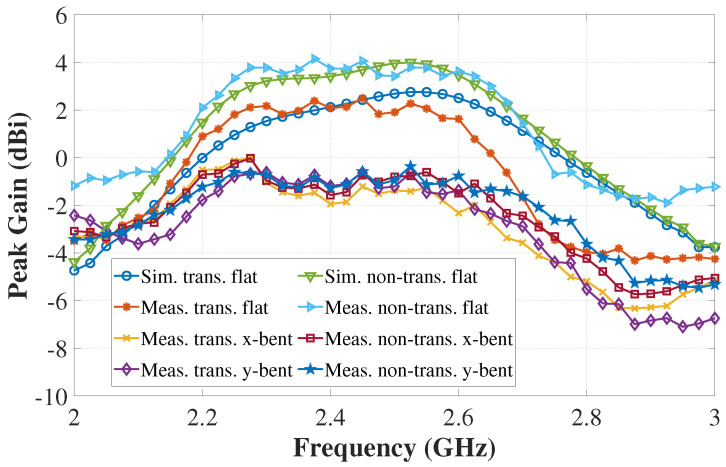
Simulated and measured peak gain vs. frequency of the transparent and non-transparent antennas.

The antenna far-field characteristics were measured in an AMS-8050 Antenna Measurement System from ETS-LINDGREN. Figure 23 displays the measurement set-up of the antenna inside the measurement chamber. The simulated and measured gains of the antennas are illustrated in Figure 24, which again shows a good agreement between the simulated and measured results of the free-space case, especially at the target operating frequency of 2.4 GHz. The discrepancy, particularly in the transparent prototype, is attributed to the fact that the transparent conductive fabric is very thin, and, hence, more vulnerable during the cutting and connecting to the SMA connector. The maximum antenna peak gain when measured flat in free space is 2.50 dBi for the transparent antenna and 4.15 dBi for the non-transparent antenna. The lower gain of the transparent antenna is expected due to the higher sheet resistance of the transparent fabric. Bending measurements over the phantom show that the gains of the antennas drop while operating on the phantom, which is the direct result of the antenna and phantom tissue coupling, particularly through the slot on the ground. Some of the radiated energy is therefore absorbed by the phantom. However, the gain performance can be improved by keeping a gap between the antenna and phantom as revealed in Figure 15.

**Figure 25:**
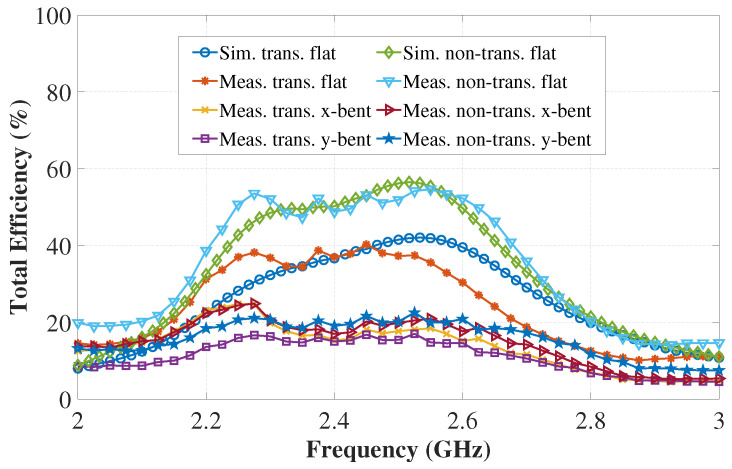
Simulated and measured total efficiency vs. frequency of the transparent and non-transparent antennas.

The simulated and measured total efficiency of the antennas are depicted in Figure 25. It can be seen that, in free-space, the transparent antenna has a measured maximum total efficiency of 42.26%, and the non-transparent antenna has a measured maximum total efficiency of 54.64%. The reduced efficiency of the transparent antenna is attributed to the higher sheet resistance of the transparent fabric. When bent over the phantom, the antenna efficiency decreases due to the power loss in the phantom as described before. By keeping a distance between the antenna and phantom, the antenna efficiency can be improved as can be seen in Figure 16.

**Figure 26:**
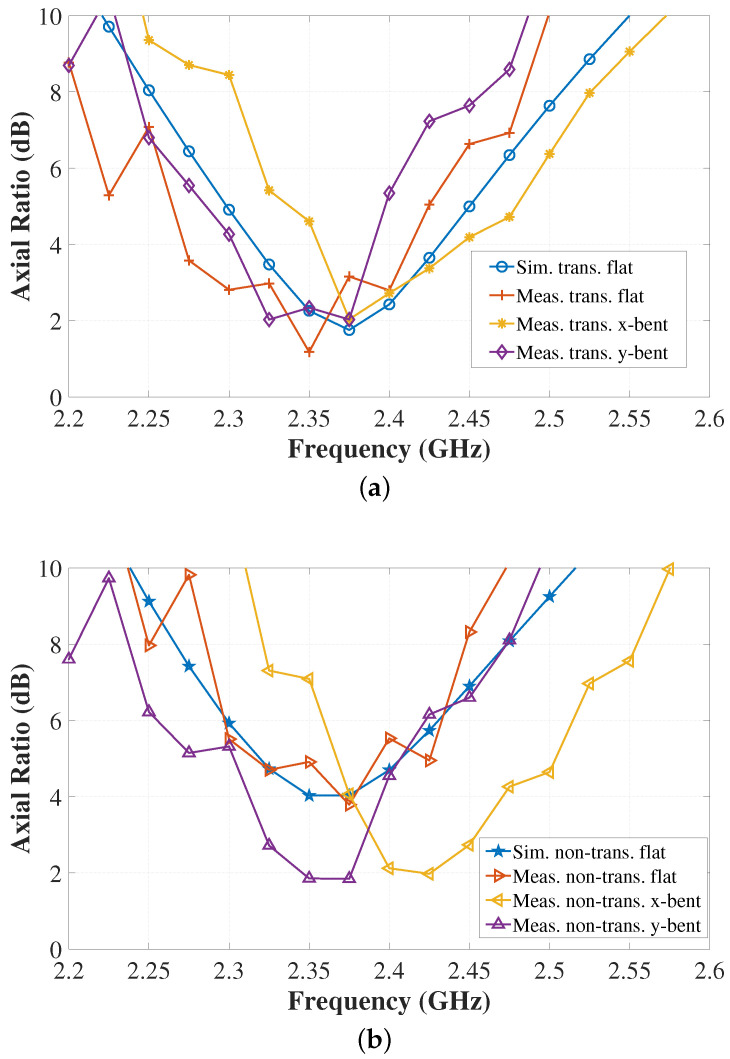
Simulated and measured axial ratio vs. frequency: (**a**) transparent antenna, (**b**) non-transparent antenna.

The simulated and measured axial ratio of the antennas in the broadside direction (θ = 0°) are provided in Figure 26. As shown before, a good agreement is apparent in the simulated and measured results of the free-space scenario. The AR results validate that the proposed transparent antenna exhibits circular polarization at the target frequency 2.4 GHz, which is still maintained even under bending on the phantom. A shift in the frequency is expected due to the antenna physical deformation upon bending. Apparently, the change in the textile conductivity and thickness does affect the AR quality of the antenna as shown in the flat non-transparent antenna result. However, upon bending on the phantom, the AR level decreases as the result of the change in the antenna physical form. The different level of change in the AR performance incurred by the non-transparent and transparent antennas upon bending is attributed to the difference in the mechanical characteristic of the resulted PDMS-textile composites.

**Figure 27:**
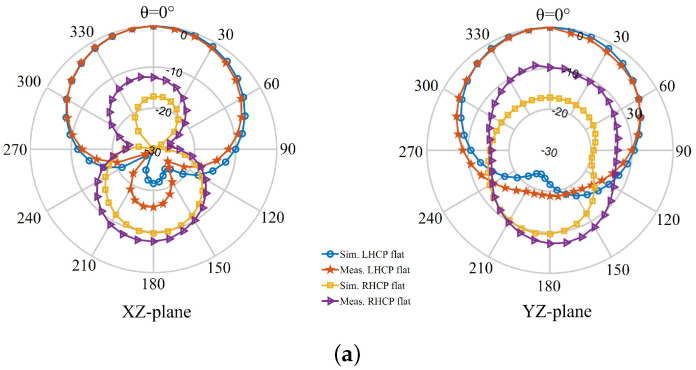

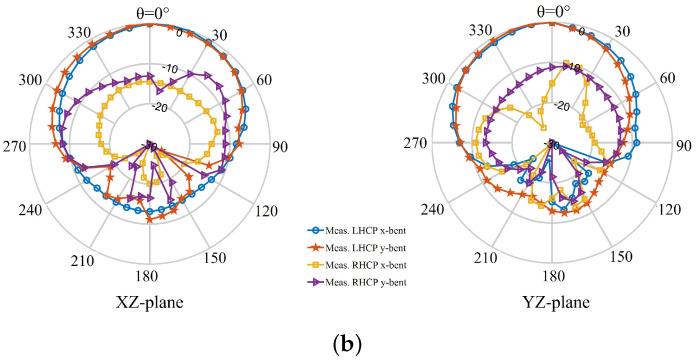
Simulated and measured far-field radiation patterns of the transparent antenna at 2.4 GHz: (**a**) unbent state, (**b**) bent state.

**Figure 28:**
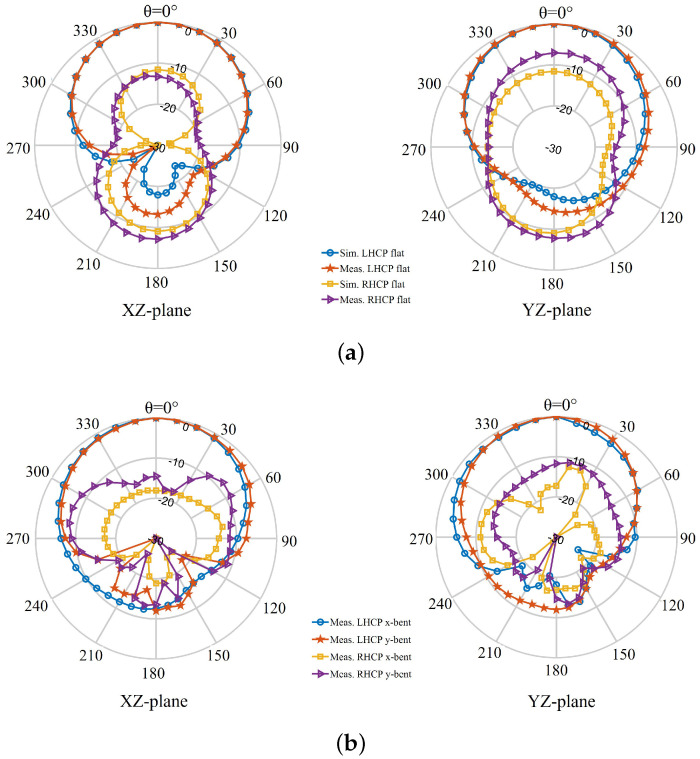
Simulated and measured far-field radiation patterns of the non-transparent antenna at 2.4 GHz: (**a**) unbent state, (**b**) bent state.

Figure 27 and Figure 28 show the far-field radiation patterns of the transparent antenna and non-transparent antenna, respectively, both in free-space and when bent over the phantom. It is revealed that the measured results closely follow the simulated patterns, particularly in the free-space case. Changes in the patterns (e.g., the level of cross-polarized components and the shape of the patterns) upon bending on the phantom are expected as the results of the change in the shape of the antennas. Considering the size and shape of the phantom, there seems to be a part of the waves, possibly from the slot in the ground, crippling to the back of the forearm phantom and contributing to the increase in the antenna back radiation for the case of antenna bent on-phantom.

## 6. Discussion

The proposed optically transparent, flexible, and circularly polarized antenna shows promising performance. The explored antenna exhibits comparable RF performance in comparison to the non-transparent flexible antenna. The low gain and efficiency of the transparent version are expected due to to the high sheet resistance of the transparent fabric, VeilShield, compared to the non-transparent, nickel-copper coated ripstop fabric. The high sheet resistance comes as a consequence of being a highly porous fabric. Despite having slightly higher sheet resistance than non-transparent nickel-copper coated ripstop fabric, VeilShield still has significantly lower sheet resistance than the transparent thin film conductors (e.g., IZTO/Ag/IZTO has a sheet resistance of 4.99 Ω/sq [12], AgHT-8 film has a sheet resistance of 8 Ω/sq [15]). The highly porous nature of VeilShield not only allows the fabric to be see-through but also to be strongly integrated with PDMS. The latter comes as a major advantage as it is certain that metallization over PDMS is notoriously difficult. With the use of VeilShield, however, it can be done easily and the bonding between PDMS and VeilShield is very strong [19], allowing for the realization of flexible transparent antenna that is mechanically robust. Maintaining strong attachment between the flexible transparent conductive and dielectric material is one of the major challenges of flexible-transparent antenna fabrication, and the explored method works as an effective solution. Moreover, the proposed method is significantly low in cost compared to the existing techniques. Thus, the demonstrated technology appears to be a highly potential technique for fabricating flexible transparent antennas. In addition, the human body is a very dynamic lossy operating environment for an antenna, and the varying position and orientation of the antenna when worn on the body might lead to a power loss associated with the polarization mismatch. The fact that the explored antenna is circularly polarized, thus it can help to maintain a reliable communication link for on-body operations. A performance comparison of the proposed flexible and transparent CP wearable antenna with some state-of-the-art CP wearable antennas is shown in Table 4. From this comparison, it is revealed that the proposed antenna is one of the lowest in terms of profile, and it is the only antenna that is concurrently flexible and transparent. The proposed antenna also shows promising 3-dB axial ratio (AR) bandwidth. The low gain and efficiency of the demonstrated antenna are attributed to the imperfect conductivity of the transparent conductive fabric and loss of PDMS. Nevertheless, considering its low profile and size, the proposed antenna exhibits decent gain and efficiency when compared to some of the reported flexible CP wearable antennas. More importantly, the highlighted properties of excellent flexibility, transparency and RF performance have not been previously demonstrated in other reported CP wearable antennas. Thus, it is ascertained that the proposed flexible and transparent CP antenna can be a strong candidate in unobtrusive wearable applications where a communication link is more prone to rapid degradation due to polarization mismatch between the transmitter and receiver. For low power applications, the demonstrated antenna will be highly efficient. In the future, further investigations will be accomplished to improve the conductivity of the transparent conductive fabric, for instance, by applying multiple metallic coating [54] or double layering of the conductive fabric [7]. These techniques will potentially reduce the performance gap between the transparent fabric-based and non-transparent fabric-based antennas.

## 7. Conclusions

The explored flexible and transparent CP antenna shows excellent performance. The antenna was rigorously tested in a flat state and on a forearm phantom; the results explicitly indicate the robustness of the antenna in bending applications. The performance of the antenna has also been compared with a similar non-transparent flexible antenna and shows comparable RF performance, demonstrating the effectiveness of the proposed transparent materials in flexible-transparent antenna manufacturing. Moreover, the SAR investigation reveals its compatibility in wearable applications. In wearable applications or other similar applications where antennas are mounted on moving objects, circular polarization maintains optimum communication performance by minimizing polarization mismatch losses. Thus, it can be explicitly expressed that the demonstrated antenna can attract significant interests in unobtrusive wearable applications.

## Figures and Tables

**Table 1:** Dimensions of the flexible and transparent CP circular patch antenna.

Parameter	Description	Value (mm)
R_*p*_	Radius of the patch	18
R_*s*_	Radius of the substrate	30
R_*g*_	Radius of the ground plane	30
R_*c*_	Radius of the slot in the ground plane	8
L_*s*_	Length of the slot in the patch	16
W_*s*_	Width of the slot in the patch	3
L_*c*_	length of the chamfer	22
L_*f*_	Length of the feed-line	12.17
W_*f*_	Width of the feed-line	5
T_*t*_	Thickness of the top PDMS cover	0.2
T_*b*_	Thickness of the bottom PDMS cover	0.2
T_*s*_	Thickness of the substrate	3

**Table 2:** Summary of the antenna performance when varying the radius of the slot of the ground plane.

**R_*c*_ (mm)**	0	4	8	12	16	20
**Res. Freq. (GHz)**	2.63	2.57	2.45	2.26	2.12	2.2
**Peak Gain (dBi)**	1.54	1.63	2.44	2.53	2.78	3.24
**Rad. Effi. (%)**	40	39.7	42.6	49.8	60.1	74.5
**F/B (dB): XZ-plane**	16.1	14.6	8.6	5	2.8	1.6
**F/B (dB): YZ-plane**	16.1	14.6	8.6	5	2.7	1.5

**Table 3:** Dielectric properties of the different tissue layers at 2.4 GHz.

Tissue	Relative Permittivity	Conductivity (S/m)
Bone	11.4	0.39
Muscle	52.73	1.74
Fat	5.28	0.1
Skin	37.88	1.44

**Table 4:** Comparison of the proposed antenna with some state-of-the-art CP wearable antennas.

Ref.	Freq. (GHz)	Footprint	Profile	Gain (dBi)	Effi. (%)	3-dB AR BW (%)	Trans.	Flex.
[6]	2.45	0.66${\lambda_{0}}^{2}$	0.03$\lambda_{0}$	6.03	62	2.18	No	Yes
[27]	6.15	0.58${\lambda_{0}}^{2}$	0.02$\lambda_{0}$	5.7	80	30.8	No	Yes
[28]	5.8	0.23${\lambda_{0}}^{2}$	0.2$\lambda_{0}$	2.1	72.6	2.93	No	No
[29]	2.45 5.8	0.123${\lambda_{0}}^{2}$	0.22$\lambda_{0}$	2.2 8.6	NA	31.4	No	No
[30]	5.16	0.05${\lambda_{0}}^{2}$	0.25$\lambda_{0}$	6.2	90	18.3	No	No
[31]	5.8	0.23${\lambda_{0}}^{2}$	0.05$\lambda_{0}$	6	80	1.4	No	Yes
[32]	5.5	0.1${\lambda_{0}}^{2}$	0.22$\lambda_{0}$	3.5	79.9	6.55	No	Yes
[33]	2.4	0.2${\lambda_{0}}^{2}$	0.045$\lambda_{0}$	3.5	58	2.4	No	No
[34]	4	0.28${\lambda_{0}}^{2}$	0.07$\lambda_{0}$	5.2	80	12.5	No	No
[35]	2.4	0.17${\lambda_{0}}^{2}$	0.045$\lambda_{0}$	5.2	79	2.72	No	Yes
[36]	2.45	0.24${\lambda_{0}}^{2}$	0.025$\lambda_{0}$	1.8	30.7	2.86	No	Yes
[37]	2.4	0.95${\lambda_{0}}^{2}$	0.03$\lambda_{0}$	4.4	34.7	2.96	No	Yes
[38]	1.575 1.621	0.27${\lambda_{0}}^{2}$	NA	6.2	70	1.23	No	Yes
This work	2.4	0.19${\lambda_{0}}^{2}$	0.028$\lambda_{0}$	2.5	42.26	4.16	Yes	Yes
